# Rapid detection of honey adulteration using machine learning on gas sensor data

**DOI:** 10.1038/s41538-025-00440-9

**Published:** 2025-05-15

**Authors:** Mehmet MİLLİ, Nursel SÖYLEMEZ MİLLİ, İsmail Hakkı PARLAK

**Affiliations:** 1 Department of Computer Engineering, Bolu Abant Izzet Baysal University, Bolu, Turkey; 2 Scientific, Industrial and Technological Application and Research Center (SITARC), Bolu Abant Izzet Baysal University, Bolu, Turkey

**Keywords:** Engineering, Risk factors

## Abstract

Honey has long been an essential component of human nutrition, valued for its health benefits and economic significance. However, honey adulteration poses a significant challenge, whether by adding sweeteners or mixing high-value single-flower honey with lower-quality multi-flower varieties. Traditional detection methods, such as melissopalynological analysis and chromatography, are often time-consuming and costly. This study proposes an artificial intelligence-based approach using the BME688 gas sensor to detect honey adulteration rapidly and accurately. The sensor captures the gas composition of honey mixtures, creating a unique digital fingerprint that can be analysed using machine learning techniques. Experimental results demonstrate that the proposed method can detect adulteration with high precision, distinguishing honey mixtures with up to 5% resolution. The findings suggest that this approach can provide a reliable, efficient, and scalable solution for honey quality control, reducing dependence on expert analysis and expensive laboratory procedures.

## Introduction

Honey, an essential part of human nutrition used in various cultures and geographies since ancient times, has great value as both a sweetener and a source of health^1^. In addition to being a natural source of energy, it supports the immune system thanks to the vitamins, minerals, and antioxidants it contains. Today, especially in developed countries, honey is gaining more popularity as a sweetener than sugar due to its numerous health benefits and low glycemic index^2^. This interest will increase exponentially, and honey will continue to be an indispensable element of human nutrition.

Honey is not only a source of food but also an important economic product worldwide. Today, honey has a strong position among its natural counterparts in the global market^3^. Honey, consumed and traded for centuries, is valuable in local and international markets today. With the recent increase in health awareness, people are turning to natural and additive-free products. This trend increases the demand for natural and organic honey, which causes the prices of quality honey to rise even more.

Considering honey’s high nutritional and economic value, health benefits, and high consumer demand, it is not surprising that it is frequently adulterated in the food sector. When difficult production conditions are added, this situation becomes a factor that threatens the reliability of honey. Therefore, the protection of quality honey and the assurance of consumer rights are critical. However, the frequent adulteration of honey risks consumer health and negatively affects market dynamics by damaging the sector’s reliability. Therefore, it is vital to protect the quality of honey and use the proper methods in inspections.

The composition of honey is characterised by the type of plant from which the bees collect nectar, climatic conditions, environmental factors, and beekeeping practices^4^. Generally, honey is divided into two categories: flower and nectar^5^. The former is obtained from flower nectar, while the latter is produced from the secretions of living plant parts left behind by plant-sucking insects^6^. Honey obtained from flower nectar has a higher economic value than that from plant parts. In addition, flower honeys are classified as single-flowered and multi-flowered honeys. Single-flowered honey is a honey class produced from a defined botanical source with distinct organoleptic properties and is more valuable than multi-flowered honey^7^. For this reason, the most adulterated honey types are single-flower honey, which has a higher economic value than others.

Adulteration of honey can be done in different ways. The most well-known of these is the addition of sweeteners such as glucose, corn syrup, and rice syrup. Another method of adulteration is the use of natural colourants or artificial flavourings. The addition of certain chemicals is also among the methods used to change the appearance or consistency of the product. The poor quality of such adulterations can be easily understood with sensory and textural analysis conducted by experts in the field. However, adulteration caused by mixing valuable and less valuable honey requires more systematic methods and different perspectives.

One of the oldest known methods for adulteration detection of honey is the determination of honey’s textural, structural, and chemical properties. However, while determining the physico-chemical properties of honey requires a comprehensive study, it can only be expected to create a framework for some types of honey^8^. Until recently, one of the most popular methods used to determine the type of honey was melissopalynological analysis, which is based on the microscopic identification of the type of pollen present in the honey^9,10^. Difficulties such as plant diversity in different regions can make it challenging to identify pollen, and pollen diversity shows seasonal changes are among the problems specific to this method and waiting to be solved.

Another method used to determine the quality of honey is gas chromatography. This method can also separate honey into its components and detect the presence of unnatural sugars or additives^11^. In addition to these methods, other techniques have recently attracted interest in determining honey adulteration. These methods may be listed as near-infrared spectroscopy^12^, Fourier transform infrared spectroscopy^13^, electrical impedance spectroscopy^14^, Raman spectroscopy^15^, nuclear magnetic resonance (NMR)^16,17^.

These methods used to detect forgery in honey are multi-stage, time-consuming, and costly. These disadvantages require expert knowledge in all stages of detecting fraud in honey. As the number of samples increases, the labour requirement will also increase. Performing the analyses may require a high level of skill and expertise. If the necessary care is not taken in the analyses, the probability of encountering erroneous results increases. In addition, many studies described above have been used to detect forgery, which involves mixing foreign substances such as corn syrup, rice syrup, and glucose into honey. For these reasons, there is an ongoing need to develop reliable, practical, faster, and more general methods and perspectives, especially for distinguishing between honeys of different floral origins, which is a more complex problem than other adulteration methods.

Sensor-based systems, which have been used in many different areas, also have the potential to be used in determining honey adulteration. However, the need for a modern sensor to make precise measurements and accurately identify the gas composition formed by different honey mixtures is one of the most significant challenges encountered in this area. In this study, the BME688 sensor, which was launched by Bosch Sensortec in 2021, was used to collect instant data on the gas concentration in the environment^18^. The BME 688 sensor can create a unique identity, that is, a digital fingerprint, of the gas concentration of its environment. This sensor, which can detect various gases in the measurement environment, is used for different purposes in many industrial applications.

The BME 688 gas sensor can detect a broad spectrum of gases, including volatile organic compounds (VOCs), volatile sulphur compounds (VSCs), and carbon monoxide and hydrogen, in the parts per billion (ppb) range. Therefore, the BME688 sensor can be customised and developed for any application scenario where VOCs and VSCs play a significant role. Some of the application areas where the BME688 sensor can be used include indoor and outdoor air quality monitoring^19,20^, food freshness detection^21^, early forest fire detection^22^, anomaly detection in critical areas and laboratory environments^23^, disease diagnosis and monitoring^24^, bad odour detection^25^, leakage detection^26^, and bacterial growth detection in industrial areas and laboratory environments^27^.

In food, timely detection of adulterated products is crucial for consumer health and safety. When the literature is examined, it is not seen that the BME 688 gas sensor is used for this purpose. In 2018, a portable electronic nose was produced using ten metal oxide sensors to detect the main components of many VOCs in peaches and total fungal contamination in fruits^28^. In 2019, an electronic nose was created using eight metal oxide sensors to detect strawberry freshness^29^. In 2021, A. Xu et al. developed a food freshness classifier for different categories of food products^30^.

In 2024, analysis and measurements were made for freshness detection on some selected vegetable and fruit samples for 50 days with the BME688 sensor^31^. In another study conducted in 2024, Dokic et al. investigated the gases released during cow milk spoilage with the BME688 gas sensor^32^. In this study, the freshness of milk was estimated by processing the data using methods such as classification and regression analysis. The results show that this approach provides a practical and cost-effective method for monitoring the quality of dairy products and detecting their freshness.

Honey adulteration detection has been extensively researched using various analytical techniques due to its significant economic and health implications. Traditional methods such as melissopalynological analysis, chromatography, and spectroscopic techniques (NIR, FTIR, Raman, and NMR) are widely employed to ascertain honey’s botanical origin and composition. These approaches can effectively identify the presence of foreign sweeteners like glucose and corn syrup and specific chemical markers indicative of adulteration. However, they often necessitate expensive laboratory equipment, highly trained personnel, and lengthy processing times, making them impractical for large-scale food quality control.

Recent studies have explored alternative techniques, including electronic nose (e-nose) technology and other sensor-based methods, to overcome these limitations. These approaches show promise for rapid and non-invasive detection of adulteration. However, most of these studies have focused on broad adulteration detection rather than addressing the more complex challenge of differentiating high-value single-flower honey from multi-flower blends. The ability to distinguish honey based on floral origin is crucial, as single-flower honey often commands higher market prices and is more susceptible to economically motivated adulteration.

Despite advancements in analytical methods, there remains a critical need for a rapid, cost-effective, and scalable solution that can be used outside specialised laboratories. Current research has not fully explored the potential of gas sensors combined with machine learning to detect honey adulteration based on VOCs and unique odour fingerprints. Gas sensors offer a promising avenue for real-time analysis by capturing the distinct chemical signatures of honey samples, enabling faster and more efficient quality control.

This study aims to bridge this gap by leveraging the BME688 gas sensor to analyse honey samples and accurately detect adulteration. To achieve this, single-flowered chestnut honey, which has a high market value, was mixed with multi-flowered honey at specific ratios, and the gas compositions of these mixtures were analysed. The BME688 sensor was used to create a digital odour fingerprint of each sample, which was then processed using machine learning algorithms to classify honey mixtures and detect adulteration.

The results of this study demonstrate that an AI-driven approach using gas sensors can provide a reliable, practical, and scalable solution for honey authentication. This method reduces reliance on traditional chemical analysis and expert interpretation and offers a faster and more efficient alternative to classical methods. Additionally, it contributes to developing AI-driven food quality assessment systems, which can be expanded to other food products in future research.

## Results

### Classification performance

Table 1 shows that with HP 504 and 414, all classification algorithms can perfectly predict the ratio of the chestnut honey mixture. The 504 or 414 profiles of the sensor matrix used can perfectly detect a resolution of 25%.

**Table 1:** CH 100, CH 75, CH 50, and CH 25 classification accuracy scores

	HP	KNN	GNB	BT	MLP	VCLF	BCLF
Matrix 1	354	1	0.99	1	1	0.99	1
	414	1	1	1	1	1	1
	504	1	1	1	1	1	1
	323	1	1	1	1	1	1
Matrix 2	354	1	1	1	0.99	0.997	1
	414	1	1	1	1	1	1
	504	1	1	1	1	1	1
	323	0.98	0.97	1	0.97	0.968	0.967

Table 2 shows that BCLF is highly accurate in detecting 5% resolution in honey mixtures when using HP 504, but it could be better. Other models, such as MLP, VCLF, or KNN, produce better accuracy scores on HP 414 and 504 data. This difference in accuracy scores might be related to differences in BAIS and our data preprocessing techniques.

**Table 2:** CH 100, CH 95, CH 90, and CH 75 classification accuracy scores

	HP	KNN	GNB	BT	MLP	VCLF	BCLF
Matrix 1	354	0.9	0.77	0.9	0.92	0.845	0.848
	414	1	1	1	1	1	0.829
	504	0.99	1	1	1	1	0.872
	323	1	1	1	1	0.997	0.802
Matrix 2	354	0.9	0.76	0.9	0.9	0.828	0.854
	414	1	1	1	0.99	0.997	0.195
	504	1	1	1	1	1	0.978
	323	0.95	0.85	0.9	0.88	0.852	0.736

Table 3 shows the accuracy scores over all eight classes. Since BCLF is limited to a maximum of four classes, its accuracy scores are not shown in Table 3. Again, HP 414 and 504 data yield the best classification accuracies.

**Table 3:** Classification accuracy scores over all eight classes

	HP	KNN	GNB	BT	MLP	VCLF
Matrix 1	354	0.95	0.88	0.9	0.92	0.891
	414	1	1	1	1	1
	504	0.99	1	1	0.96	1
	323	1	1	1	0.92	0.997
Matrix 2	354	0.92	0.88	0.9	0.85	0.885
	414	0.96	1	1	0.92	0.999
	504	1	1	1	0.98	1
	323	0.9	0.92	1	0.92	0.926

### Regression performance

Table 4 shows regression models’ mean absolute error (MAE) scores. MAE is calculated using the formula given in Eq. (1), where ${\hat{y}}_{i}$ is the model’s prediction for the ith sample, and ${y}_{i}$ is the actual value. The lower the MAE score, the higher the model’s accuracy.

1 $${MAE}=\frac{1}{N}\mathop{\sum }\limits_{i=1}^{N}\left|{\hat{y}}_{i}-{y}_{i}\right|$$

MAE formula^33^.

**Table 4:** MAE scores of regression models

	HP	LR	GBR	EN	SGDR	BAR
Matrix 1	354	2.315	0.087	5.097	3.483	5.25
	414	2.007	0.006	4.933	3.216	5.39
	504	1.309	0.034	3.65	1.721	4.16
	323	2.19	0.067	4.597	4.184	3.68
Matrix 2	354	2.38	0.176	4.967	4.403	8
	414	1.512	0.012	4.513	3.336	25.76
	504	1.44	0.025	3.93	2.735	3.59
	323	2.124	0.366	6.365	3.718	8.01

The table shows that most regression models score best with HP 504. Although a direct comparison of BAR and other models is impossible for the current scenario, we can say that among our regressors, GBR performs the best on the regression task.

Lastly, Table 5 shows the *R*^2^ scores of all regression models except BAR since BAIS does not support *R*^2^ score calculation. Again, GBR shows the best accuracy in the regression task. Additionally, when looking at the scores of other models, it is seen that the best profile for the regression task is HP 504, followed by HP 414.

**Table 5:** *R*^2^ scores of regression models

	HP	LR	GBR	EN	SGDR
Matrix 1	354	0.994	1	0.976	0.987
	414	0.995	1	0.975	0.988
	504	0.998	1	0.987	0.996
	323	0.994	1	0.978	0.983
Matrix 2	354	0.993	1	0.977	0.98
	414	0.997	1	0.98	0.989
	504	0.997	1	0.984	0.993
	323	0.994	0.999	0.958	0.984

## Discussion

When all the results are examined, it can be concluded that the BME688 sensor is a reliable and effective tool for detecting adulteration in chestnut honey. The study demonstrated that even 5% adulteration can be accurately identified, provided that the appropriate heater profile (HP) is selected, with HP 504 and HP 414 showing the best performance. Additionally, highly accurate regression models allow food inspectors to determine the proportion of chestnut honey in a mixture without being limited to discrete classification levels. The system developed in this study provides a faster and more practical alternative to traditional honey authentication methods such as melissopalynology, gas chromatography, and NMR. It produces reliable results in approximately 20 min. Unlike conventional approaches, this method reduces dependency on expert interpretation and provides a scalable AI-driven solution.

Despite its advantages, the study also has certain limitations. Model training, data preprocessing, and parameter optimisation in AI-based systems require complex and time-consuming processes; however, these steps are necessary to ensure high accuracy, robustness, and generalizability. Moreover, once the framework is established, subsequent classifications can be performed quickly and efficiently. The BME688 sensor is sensitive to environmental factors such as humidity and temperature, requiring careful calibration and controlled measurement conditions. Additionally, honey’s chemical composition can vary due to seasonal and geographical factors, similar to sensory and chemical analysis challenges. Therefore, expanding the dataset with different honey types from various regions is crucial to improve model generalizability. Otherwise, the false positives/negatives rate may increase significantly and negative results may occur in real-world applications. Furthermore, sensor-based methods are not yet standardised to the same extent as traditional chemical analyses, necessitating further validation and refinement for widespread adoption.

The main reason for the complexity and time-consuming nature of this study is the comprehensive evaluation of multiple machine learning models and HPs, which establishes a robust foundation for future research. Unlike integrated electronic nose systems, processing gas sensor data with AI enhances classification accuracy, enabling precise differentiation of honey’s floral origin. In the long term, this research contributes to the development of optimised, rapid, and scalable AI solutions for food fraud detection, paving the way for more reliable and practical applications in both academic and industrial settings. Future work will focus on expanding the dataset with a wider range of honey samples and further improving the detection capabilities of the BME688 sensor for different types of honey adulteration.

## Methods

### Honey samples

To complicate the level of adulteration and to understand the distinguishability of machine learning approaches, a type of honey that is easier to obtain and cheaper than chestnut honey was used in the adulteration instead of glucose syrup or sweetener. The adulteration of honey prepared this way will be challenging to understand, even with expert sensory analyses. Within the scope of the study, chestnut honey, which is more difficult to obtain and has a higher market value than other types of honey, was adulterated with flower honey. For chestnut honey and other flower honey used as adulterated material, products of a reliable brand, frequently preferred in Turkey, were preferred. The products were obtained from a reliable chain store that is frequently preferred in Turkey. The producer company of the acquired chestnut honey has a certificate of conformity to Turkish standards numbered 042561-TSE-04/02 from the Turkish Standards Institute. In the study, comprehensive laboratory analyses were conducted on chestnut honey and flower honey used for adulteration^34,35^. According to these reports, the pollen content of chestnut honey was determined to be 82%.

### Sensor matrix and configuration

**Fig. 1:**
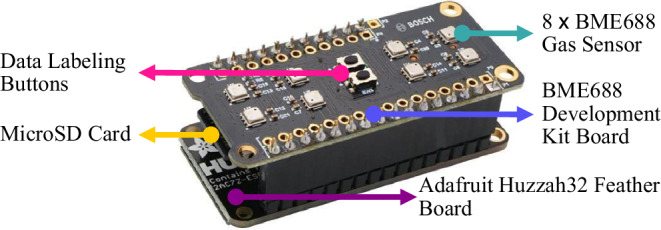
BME688 sensor matrix and its components. Each metallic square object at the top of the matrix is a BME688 sensor. This figure is adapted from the sensor development kit manual^36^.

Instead of performing the measurement operations with a single BME 688 sensor, they were performed with matrices containing eight sensors each. The sensor matrix used for the measurement operation is shown in Fig. 1. Each sensor on the sensor matrix can collect data independently of each other in different profiles. Therefore, the most significant advantage of using a sensor matrix is that it allows the reaction of different profiles to the gas concentration in the environment to be evaluated without the need to prepare a new measurement environment. Sensor-based measurement operations may need to be verified with equivalent or similar sensors. Another advantage of using a matrix is that there are enough sensors on the development board to verify each other. Sixteen sensors on two different BME 688 sensor development boards were used for the measurement operations.

**Fig. 2:**
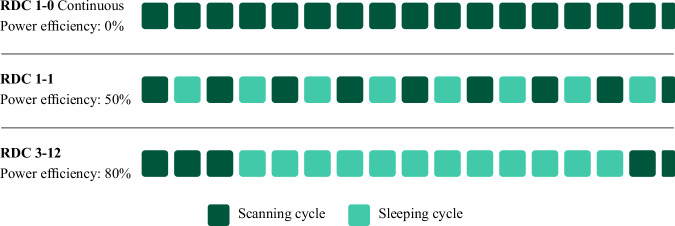
Some of the available RDC scanning and sleep times, along with related power efficiency in BAIS.

The embedded system coding of the BME688 sensors in the development kit was performed using the AI-Studio software developed by Bosch. Bosch AI Studio (BAIS) has 17 different HP designed to detect different gas properties in different application situations of the BME688 sensor. Additionally, it features 16 different duty cycle (RDC) profiles, developed to optimise the power consumption of the sensor matrices. Although these ready-made heaters and duty cycle profiles provide convenience for embedded system coding, they may have disadvantages, such as needing more flexibility for more specific applications. The different RDC operation and sleep times, along with the resulting power efficiency, are shown in detail in Fig. 2.

The energy consumption of the sensor matrices can be significantly reduced by utilising different RDC profiles and placing them in sleep mode for specific periods. In cases where it is impossible to provide continuous energy to the sensors, energy consumption can be reduced by up to 80%. However, it is observed that using RDC modes increases the energy efficiency of the sensors while significantly reducing the scanning performance. This decrease leads to longer response times for the sensors and a decrease in the application’s sensitivity. For this reason, it is crucial to choose an appropriate RDC profile that will carefully balance the scanning performance and power consumption. Since the studies were carried out in a laboratory environment, no difficulty was experienced in providing energy to the sensor nodes. In this context, the sensors were never put into sleep mode, and the coding was done using the RDC-1-0 duty cycle profile. Thus, the scanning performance of the BME 688 sensors was maintained.

**Fig. 3:**
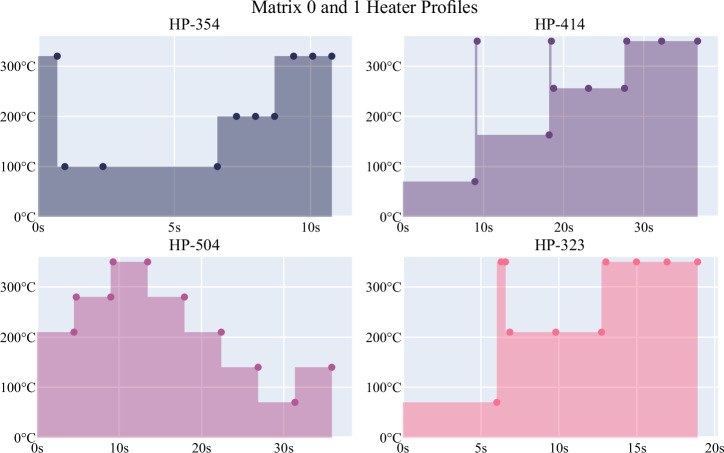
HPs of matrices. Each HP shows the sensors to different temperatures at different times.

In this study, to verify the data obtained by each sensor, groups of two consecutive sensors in each sensor matrix were formed, and embedded system coding was performed using the same HPs. In addition, to determine the best HP profile that will provide fast and sensitive detection of honey adulteration, embedded system coding was performed with different HPs for each group. The HPs coded to the sensor groups are HP-354, HP-323, HP-414, and HP-504 profiles, respectively. The HPs coded for the four groups of sensors on the sensor matrices are shown in detail in Fig. 3.

### Experimental setup

**Fig. 4:**
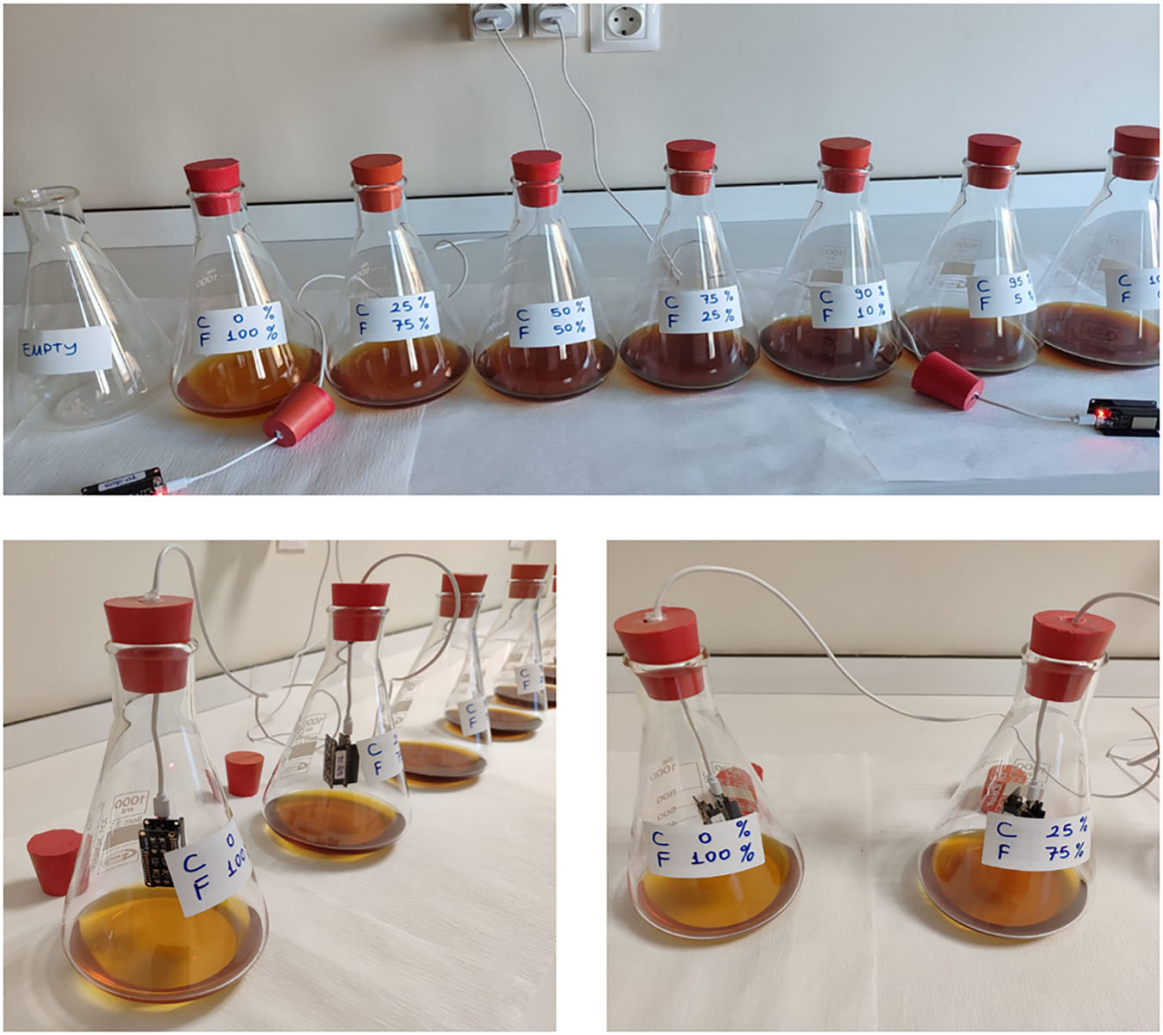
Laboratory environment: honey mixtures, sensor placements. Each Erlenmeyer flask has a label indicating the honey mixture inside.

Chestnut honey, which is a single-flower honey with distinct organoleptic properties and sold at higher prices than other honey in the market, was used in the study. Multi-flower honey, which is much more affordable, was used as an adulterant. These honeys were mixed in various proportions to create seven honey mixtures with different concentrations. The chestnut honey and flower honey ratios in these mixtures were determined as (1) 100-0, (2) 95-5, (3) 90-10, (4) 75-25, (5) 50-50, (6) 25-75, and (7) 0-100, respectively. The setup created for experiments and measurements in the laboratory environment is shown in Fig. 4.

To increase the viscosity of the chestnut and flower honeys used in the analysis, distilled water was added to 10% of their weight. After this process, mixing was carried out for 10 min at 45 °C in a heated magnetic stirrer (IKA RTC Basic, Germany). Thus, the honey samples were provided with a more homogeneous structure. Then, the weighing process was carried out in a way that the predetermined % mixing ratios would be 200 g (*w*/*w*) in total. Each sample was placed in a different 1000 ml Erlenmeyer flask.

The power cables of the sensor matrices were passed through rubber stoppers to keep them stable during the measurements. The sensor matrices were placed approximately 3 cm above the samples in the Erlenmeyer flasks. The Erlenmeyer flasks, with wide bases and narrowing as they rose, ensured that the gas concentrations from the honey mixtures were concentrated in the area where the sensor was located. In this way, the sensors detected the characteristic gas concentrations of each honey mixture more clearly.

The sensor matrices were operated at room conditions for 2 h before starting the measurements to ensure the sensors reached a steady state. The measurements of the gas compositions of the honey mixtures were carried out between 07:00 and 17:00 on 24.10.2024. The sensor matrices were left in each container containing different honey mixtures for 30 min to allow them to take measurements. After each sample measurement, the sensors were operated at room conditions for 30 min to return to a steady state and remove gas residues from the previous mixture from their membranes. After the sensors reached a steady state, they were placed in the next Erlenmeyer flask to measure the concentration of the following gas mixture. This cycle was continued until sufficient sensor measurements were obtained from all honey mixtures. The experiments were carried out in a climate-controlled laboratory environment at a constant temperature of 23 °C. When the relative humidity and temperature data recorded by the sensor matrices used in addition to the resistance values of the gas composition were examined at the end of the experiments, it was seen that there was no significant change in the relative humidity and temperature values during the experimental process that would affect the results of the study.

### Dataset and data validation

**Fig. 5:**
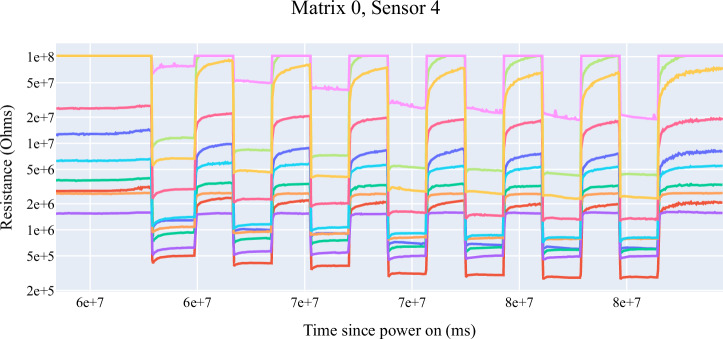
Graph of resistance values collected by sensor 4 of matrix 0. The low areas in the graph indicate the moments when the sensor is measuring honey.

Two different data sets were created using the data obtained from the sensor matrices. The first matrix, called Sensor Matrix 0, contains 3967 cycles. Only one cycle was dropped in this matrix during the data collection process. The total data collected by this matrix is 39666. The second matrix contains a total of 3826 cycles. During data collection, it was observed that there was only one cycle drop, just like in the first matrix. The total number of data collected by this matrix is 38255. Since the same HPs are loaded on both matrices, the total cycle and data numbers are expected to be close to each other. As an example, Fig. 5 shows the gas resistance data measured by the BME 688 sensor number 4 of Sensor Matrix 0.

In the graph of data collected by a BME 688 sensor, any imaginary vertical line intersects the graph at ten points. These ten points represent the digital odour fingerprint of a sample at a given time. In other words, it represents the unique barcode or QR code of the gas composition in the measurement environment at that moment. When the measurements are examined, it is seen that as the chestnut honey ratio in the mixture decreases and the flower honey ratio increases, the resistance of the gas concentration in the environment decreases. This information will be beneficial in determining the ratios of honey in the mixtures.

**Fig. 6:**
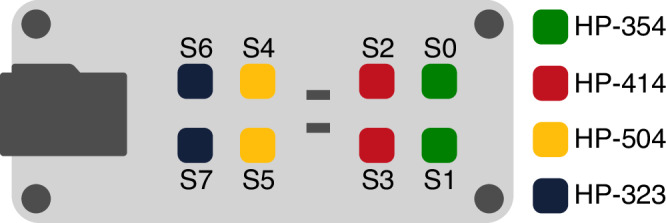
Embedded system coding of sensor matrices was performed with the same HPs to verify each other.

Proving the accuracy of sensor data is of critical importance in ensuring the reliability of sensor studies. Accurate and reliable sensor data directly affects the validity of the findings obtained, which in turn determines the success of the results in the application phase. In addition, verification processes help to ensure a secure and accurate data flow independent of all external factors by minimising calibration errors and other potential deviations of sensors. One of the most commonly used verification methods in the literature is equivalent sensor or device measurements. Fig. 6 shows the HP profiles loaded on BME 688 sensors on sensor matrices.

In this study, eight sensors in the matrices were grouped in pairs to verify sensor data. As seen in Fig. 6, the same profiles were loaded into each group. In addition, a second sensor matrix was used to verify data across the sensor matrix. Again, in this matrix, sensors were divided into groups of 2, and the profiles in matrix 0 were loaded as they were. In this way, both the data measured by sensors on one matrix were verified by a second sensor and verification was also provided by another sensor on another matrix. After the data collection stage, it was observed that sensors loaded with the same profiles on Sensor Matrix 0 and Sensor Matrix 1 produced similar data graphs.

### Data preprocessing

**Fig. 7:**
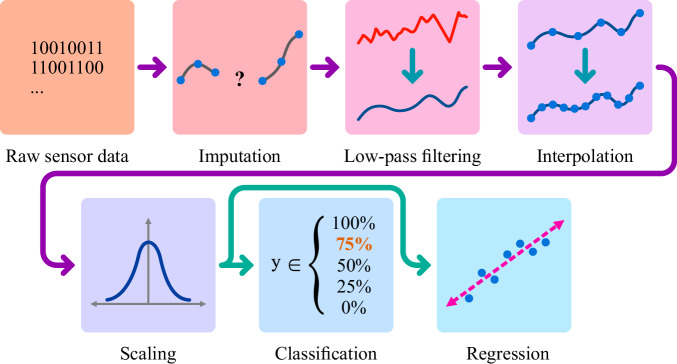
The flow of data from sensors to machine learning models. Raw data undergoes several preprocessing steps before becoming a suitable input for the machine learning models.

The data obtained from the sensor matrices underwent a series of pre-processing steps before being used in machine learning algorithms. The dataset had 23 missing sensor measurements, whereas the total number of successful measurements was 701,914. So, the ratio of missing data to successfully measured data is 32e-6, which is very low. As seen in Fig. 7, the missing data were imputed first. Each missing value was imputed by taking the arithmetic means of its preceding and succeeding neighbours.

**Fig. 8:**
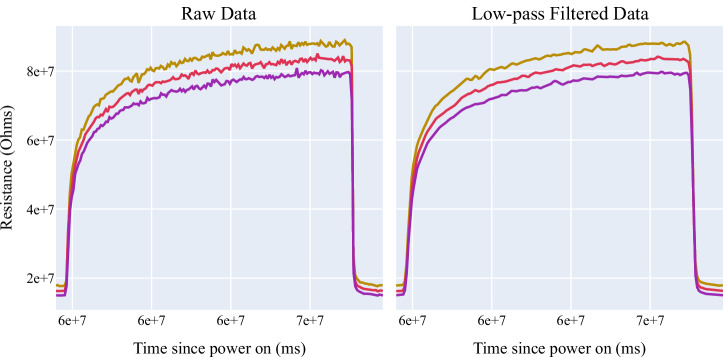
A portion of the raw data (left) and low-pass filtered and smoothed data (right).

The second step in our data pre-processing is applying a low-pass filter to the now-imputed data. This filtering out of noise in data leaves more efficient and smoother curves. To perform smoothing, a digital Butterworth filter was applied with these parameters: sampling rate = 50, cutoff = 10, and order = 1. The filter was also applied both forward and backwards to prevent phase shifting. The effects of applying a low-pass filter can be seen in Fig. 8.

Sensor measurements in a single cycle are not aligned in the time domain. This is due to the design of the sensors. Sensors perform measurements according to their HPs. All HPs operate their sensors to reach a specific temperature at a specific time to take successive measurements. Consequently, even though they belong to the same cycle, the ten captured measurements are not perfectly aligned on the time axis. Univariate spline functions were fitted to the sensor readings to align the samples within the same cycle in the time domain. This process produces spline functions that estimate sensor readings at any desired time.

The final step in data pre-processing is scaling. Captured gas resistance values are in the range of 1e5 and 1e8, which is significantly large. We first applied the natural logarithm to all gas resistance readings to reduce this range. Additionally, some machine learning algorithms perform better with standardised data. So, as the last step of pre-processing, we applied standard scaling, as shown in Eq. (2), to the data to enhance the accuracy of the machine learning models. The pre-processed data was then used to train classification and regression models.

2 $$z=\,\frac{x-\mu }{\sigma }$$

Standard scaling formula: $x$ is the feature matrix, $\mu$ is the mean of $x$, $\sigma$ is the standard deviation of $x$, and $z$ is the scaled feature matrix.

This study did not utilise feature selection or dimensionality reduction methods because they were unnecessary, given the dataset’s characteristics. Since HPs were assigned to sensors in pairs, and each measurement cycle contained 10 features, each sample used to train the machine learning models had only 20 features—these dataset characteristics yielded relatively low-dimensional data that did not pose considerable computational challenges. Besides, the dataset was not particularly large, and training times remained reasonable even with all features included. Also, the machine learning models achieved high accuracy scores without employing any feature reduction techniques.

### Classification

There are eight classes in the dataset which are air, 100% chestnut honey—0% flower honey mixture (CH 100), 95% chestnut honey—5% flower honey mixture (CH 95), 90% chestnut honey—10% flower honey mixture (CH 90), 75% chestnut honey—25% flower honey mixture (CH 75), 50% chestnut honey—50% flower honey mixture (CH 50), 25% chestnut honey—75% flower honey mixture (CH 25), and 0% chestnut honey—100% flower honey mixture (CH 0). However, as a significant drawback, the BAIS application limits the maximum number of classes to 4. We performed several classification experiments to compare BAIS’s classification model with other models outside of BAIS. In all classification experiments, classification algorithms are K-Nearest Neighbours (KNN), Gaussian Naïve Bayes (GNB), Bagging Tree (BT), Multi-layer Perceptron (MLP), Voting Classifier (VCLF), and BAIS Classifier (BCLF). GNB and BT models are chosen for computational efficiency. On the other hand, KNN, GNB, and BT are highly interpretable. MLP can capture complex patterns in the data at the cost of efficiency and interpretability. Finally, VCLF can improve the generalisation and reduce bias by combining multiple models.

The first experiment was performed in the CH 100, CH 75, CH 50, and CH 25 classes. The classifier accuracy results of this experiment are shown in Table 1. A classifier’s accuracy is calculated by dividing the number of correct predictions by the total number of predictions. Therefore, the maximum score of any model’s accuracy can be 1, whereas the minimum can be 0. All classifiers’ accuracies are obtained using 5-fold cross-validation except BCLF. BAIS, as another drawback, does not support the functionality of performing N-fold cross-validation. BAIS application BCLF’s accuracy score is calculated over a randomly selected 20% test set. Although the functionalities provided by BAIS are serviceable, the application has some limitations, such as the lack of cross-validation functionality and the restriction to a maximum of four different classes for classification. Due to these limitations, the authors have used other algorithms outside the BAIS environment.

The second experiment was performed over the CH 100, CH 95, CH 90, and CH 75 classes. In this experiment, chestnut honey mixture percentages were selected to be as close to each other as possible. This experiment aimed to determine the accuracy of flower honey detection resolution. The results of this experiment are given in Table 2.

The final classification experiment was performed on all eight classes. As mentioned, BAIS limits the number of classes to 4, so BCLF accuracy scores for this experiment are unavailable. The results of the final classification experiment are given in Table 3.

### Regression

Unlike classification models, which can make categorical predictions, regression models can calculate continuous dependent variables. As such, accurate regression models can predict any arbitrary ratio of chestnut honey in a mixture. In this study, we trained several regression models and compared their performances according to their MAE and *R*^2^ scores. Trained regression algorithms were linear regression (LR), gradient boosting regression (GBR), elastic net (EN), stochastic gradient descent regression (SGDR), and BAIS’s regression model (BAR). GBR can handle high data complexity, while LR and SGDR are computationally efficient. EN is a moderately efficient model that handles medium data complexity.

BAIS can train regression models and supports several types of accuracy measurement metrics. However, it does not calculate the *R*^2^ scores of regression models. In Table 4, MAE scores of all regression models are given. However, since knowing BAIS’s data pre-processing and scaling methods is unattainable, a direct comparison of BAR and other regression models is impossible. In Table 5, *R*^2^ scores of regression models except BAR are presented.

## Data Availability

Data and source code is available at https://github.com/ihpar/cn_honey.

## Code availability

The source code described and used in this article is available and can be accessed at https://github.com/ihpar/cn_honey.
